# *N*-(3,4-Difluoro­phen­yl)-2,2-diphenyl­acetamide

**DOI:** 10.1107/S1600536812014675

**Published:** 2012-04-13

**Authors:** Hoong-Kun Fun, Chin Wei Ooi, Prakash S. Nayak, B. Narayana, B. K. Sarojini

**Affiliations:** a X-ray Crystallography Unit, School of Physics, Universiti Sains Malaysia, 11800 USM, Penang, Malaysia; b Department of Studies in Chemistry, Mangalore University, Mangalagangotri 574 199, India; c Department of Chemistry, P. A. College of Engineering, Nadupadavu, Montepadavu, PO, Mangalore 574 153, India

## Abstract

In the title compound, C_20_H_15_F_2_NO, the mean plane of the acetamide group makes dihedral angles of 88.26 (6), 78.30 (7) and 9.83 (6)° with the two terminal benzene rings and difluoro-substituted benzene ring, respectively. One F atom is disordered over two orientations rotated by 180°, with a site-occupancy ratio of 0.557 (2):0.443 (2). An intra­molecular C—H⋯O hydrogen bond generates an *S*(6) ring motif. In the crystal, mol­ecules are linked *via* N—H⋯O hydrogen bonds into chains along the *c* axis. The crystal structure is further consolidated by C—H⋯π inter­actions.

## Related literature

For the structural similarity of *N*-substituted 2-aryl­acetamides to the lateral chain of natural benzyl­penicillin, see: Mijin & Marinkovic (2006 ▶); Mijin *et al.* (2008 ▶). For the coordination abilities of amides, see: Wu *et al.* (2008 ▶, 2010 ▶). For hydrogen-bond motifs, see: Bernstein *et al.* (1995 ▶). For related structures, see: Praveen *et al.* (2011*a* ▶,*b* ▶,*c* ▶); Fun *et al.* (2011*a* ▶,*b* ▶). For bond-length data, see: Allen *et al.* (1987 ▶). For the stability of the temperature controller used in the data collection, see: Cosier & Glazer (1986 ▶).
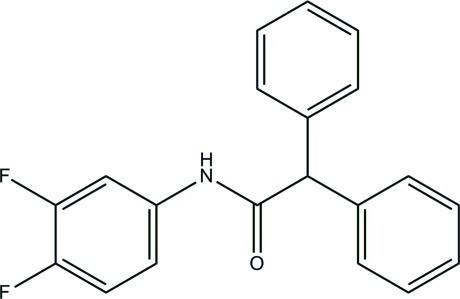


## Experimental

### 

#### Crystal data

C_20_H_15_F_2_NO
*M* *_r_* = 323.33
Monoclinic, 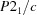

*a* = 9.9756 (2) Å
*b* = 18.0181 (3) Å
*c* = 9.8107 (2) Å
β = 117.064 (1)°
*V* = 1570.29 (5) Å^3^
*Z* = 4
Mo *K*α radiation
μ = 0.10 mm^−1^
*T* = 100 K
0.54 × 0.41 × 0.37 mm

#### Data collection

Bruker SMART APEXII CCD area-detector diffractometer
Absorption correction: multi-scan (*SADABS*; Bruker, 2009 ▶) *T*_min_ = 0.947, *T*_max_ = 0.964
30289 measured reflections
6276 independent reflections
4829 reflections with *I* > 2σ(*I*)
*R*_int_ = 0.028

#### Refinement

*R*[*F* ^2^ > 2σ(*F* ^2^)] = 0.052
*wR*(*F* ^2^) = 0.130
*S* = 1.03
6276 reflections
231 parameters
H atoms treated by a mixture of independent and constrained refinement
Δρ_max_ = 0.35 e Å^−3^
Δρ_min_ = −0.22 e Å^−3^

### 

Data collection: *APEX2* (Bruker, 2009 ▶); cell refinement: *SAINT* (Bruker, 2009 ▶); data reduction: *SAINT*; program(s) used to solve structure: *SHELXTL* (Sheldrick, 2008 ▶); program(s) used to refine structure: *SHELXTL*; molecular graphics: *SHELXTL*; software used to prepare material for publication: *SHELXTL* and *PLATON* (Spek, 2009 ▶).

## Supplementary Material

Crystal structure: contains datablock(s) global, I. DOI: 10.1107/S1600536812014675/rz2732sup1.cif

Structure factors: contains datablock(s) I. DOI: 10.1107/S1600536812014675/rz2732Isup2.hkl

Supplementary material file. DOI: 10.1107/S1600536812014675/rz2732Isup3.cml

Additional supplementary materials:  crystallographic information; 3D view; checkCIF report

## Figures and Tables

**Table 1:** Hydrogen-bond geometry (Å, °) *Cg*1 and *Cg*2 are the centroids of the C1–C6 and C8–C13 rings, respectively.

*D*—H⋯*A*	*D*—H	H⋯*A*	*D*⋯*A*	*D*—H⋯*A*
N1—H1*N*1⋯O1^i^	0.881 (15)	2.088 (16)	2.9134 (11)	155.7 (14)
C13—H13*A*⋯*Cg*1^ii^	0.95	2.98	3.7633 (11)	140
C16—H16*A*⋯*Cg*2^iii^	0.95	2.82	3.5998 (15)	140
C20—H20*A*⋯O1	0.95	2.29	2.8878 (14)	120

Symmetry codes: (i) 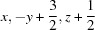; (ii) 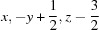; (iii) 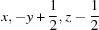.

**Fig. 1.**
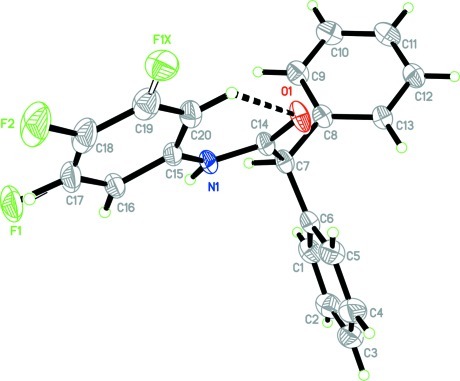
The molecular structure of the title compound, showing 50% probability displacement. An intramolecular hydrogen bond is shown a a dashed line. Bonds invoving the minor component of disorder are shown as open bonds.

**Fig. 2.**
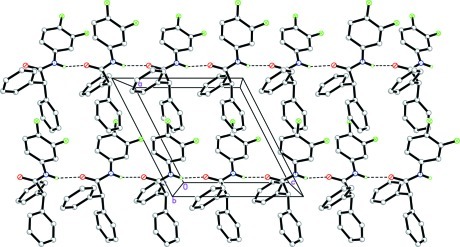
The crystal packing of the major component of the title compound, viewed along the b axis. H atoms not involved in intermolecular hydrogen interactions (dashed lines) and the minor components of disorder have been omitted for clarity.

## supplementary crystallographic information

### Comment

*N*-Substituted 2-arylacetamides are very interesting compounds because of their structural similarity to the lateral chain of natural benzylpenicillin (Mijin & Marinkovic, 2006; Mijin *et al.*, 2008). Amides are also used as ligands due to their excellent coordination abilities (Wu *et al.*, 2008, 2010). The crystal structures of some acetamide derivatives *viz*. *N*-(4-chloro-1,3-benzothiazol-2-yl)-2-(3-methylphenyl)acetamide monohydrate, *N*-(3-chloro-4-fluorophenyl)-2,2-diphenylacetamide and *N*-(3-chloro-4-fluorophenyl)-2-(naphthalen-1-yl)acetamide (Praveen *et al.*, 2011*a*,*b*,*c*) have been recently reported. In continuation of our work on the synthesis of amides (Fun *et al.*, 2011*a*,*b*), we report herein the crystal structure of the title compound (I).

The molecular structure of the title compound (I) is shown in Fig. 1. The mean plane of the acetamide group (C7/C14/N1/O1) makes dihedral angles of 88.26 (6), 78.30 (7) and 9.83 (6)° with the two terminal benzene rings (C1–C6 and C8–C13) and difluoro-substituted benzene ring (C15–C20), respectively. The F1 fluorine atom is disordered over two orientations rotated by 180° with a site-occupancy ratio of 0.557 (2):0.443 (2). The molecular structure is stabilized by an intramolecular C20—H20A···O1 hydrogen bond which generates an S(6) ring motif (Bernstein *et al.*, 1995). The bond lengths (Allen *et al.*, 1987) and angles are within normal ranges and are comparable to those found in related structures (Praveen *et al.*, 2011*a*,*b*,*c*; Fun *et al.*, 2011*a*,*b*). In the crystal structure (Fig. 2), the molecules are linked *via* intermolecular N1—H1N1···O1 hydrogen bonds (Table 1) into chains along the *c* axis. The crystal structure is further consolidated by C—H···π interactions (Table 1), involving the C1–C6 ring (centroid *Cg* 1) and C8—C13 ring (centroid *Cg* 2).

### Experimental

Diphenylacetic acid (0.212 g, 1 mmol), 2,6-difluoroaniline (0.1 ml, 1 mmol) and 1-ethyl-3-(3-dimethylaminopropyl)-carbodiimide hydrochloride (1.0 g, 0.01 mol) were dissolved in dichloromethane (20 mL). The mixture was stirred in presence of triethylamine at 273 K for about 3 h, then was poured into 100 ml of ice-cold aqueous hydrochloric acid with stirring, and extracted thrice with dichloromethane. The organic layer was washed with a saturated NaHCO_3_ solution and brine solution, dried and concentrated under reduced pressure to give the title compound (I). Single crystals were grown from a methylene chloride/*N*,*N*-dimethyl formamide mixture (1:1 *v*/*v*) by the slow evaporation method (*m.p.*: 403–405 K).

### Refinement

Atom H1N1 was located in a difference Fourier map and refined freely [N–H = 0.881 (15) Å]. The remaining H atoms were positioned geometrically and refined using a riding model with *U*_iso_(H) = 1.2 *U*_eq_(C) (C—H = 0.95–1.00 Å). The fluorine atom is disordered over two positions with refined site-occupancy ratio of 0.557 (2):0.443 (2).

### Crystal data

**Table:**

C_20_H_15_F_2_NO	*F*(000) = 672
*M**_r_* = 323.33	*D*_x_ = 1.368 Mg m^−^^3^
Monoclinic, *P*2_1_/*c*	Mo *K*α radiation, λ = 0.71073 Å
Hall symbol: -P 2ybc	Cell parameters from 9889 reflections
*a* = 9.9756 (2) Å	θ = 2.3–33.5°
*b* = 18.0181 (3) Å	µ = 0.10 mm^−^^1^
*c* = 9.8107 (2) Å	*T* = 100 K
β = 117.064 (1)°	Block, colourless
*V* = 1570.29 (5) Å^3^	0.54 × 0.41 × 0.37 mm
*Z* = 4	

### Data collection

**Table:**

Bruker SMART APEXII CCD area-detector diffractometer	6276 independent reflections
Radiation source: fine-focus sealed tube	4829 reflections with *I* > 2σ(*I*)
Graphite monochromator	*R*_int_ = 0.028
φ and ω scans	θ_max_ = 33.8°, θ_min_ = 2.3°
Absorption correction: multi-scan (*SADABS*; Bruker, 2009)	*h* = −15→15
*T*_min_ = 0.947, *T*_max_ = 0.964	*k* = −21→28
30289 measured reflections	*l* = −15→15

### Refinement

**Table:**

Refinement on *F*^2^	Primary atom site location: structure-invariant direct methods
Least-squares matrix: full	Secondary atom site location: difference Fourier map
*R*[*F*^2^ > 2σ(*F*^2^)] = 0.052	Hydrogen site location: inferred from neighbouring sites
*wR*(*F*^2^) = 0.130	H atoms treated by a mixture of independent and constrained refinement
*S* = 1.03	*w* = 1/[σ^2^(*F*_o_^2^) + (0.0529*P*)^2^ + 0.484*P*] where *P* = (*F*_o_^2^ + 2*F*_c_^2^)/3
6276 reflections	(Δ/σ)_max_ = 0.001
231 parameters	Δρ_max_ = 0.35 e Å^−^^3^
0 restraints	Δρ_min_ = −0.22 e Å^−^^3^

### Special details

**Table:**

Experimental. The crystal was placed in the cold stream of an Oxford Cryosystems Cobra open-flow nitrogen cryostat (Cosier & Glazer, 1986) operating at 100.0 (1) K.
Geometry. All e.s.d.'s (except the e.s.d. in the dihedral angle between two l.s. planes) are estimated using the full covariance matrix. The cell e.s.d.'s are taken into account individually in the estimation of e.s.d.'s in distances, angles and torsion angles; correlations between e.s.d.'s in cell parameters are only used when they are defined by crystal symmetry. An approximate (isotropic) treatment of cell e.s.d.'s is used for estimating e.s.d.'s involving l.s. planes.
Refinement. Refinement of *F*^2^ against ALL reflections. The weighted *R*-factor *wR* and goodness of fit *S* are based on *F*^2^, conventional *R*-factors *R* are based on *F*, with *F* set to zero for negative *F*^2^. The threshold expression of *F*^2^ > σ(*F*^2^) is used only for calculating *R*-factors(gt) *etc*. and is not relevant to the choice of reflections for refinement. *R*-factors based on *F*^2^ are statistically about twice as large as those based on *F*, and *R*- factors based on ALL data will be even larger.

### Fractional atomic coordinates and isotropic or equivalent isotropic displacement parameters (Å^2^)

**Table:**

	*x*	*y*	*z*	*U*_iso_*/*U*_eq_	Occ. (<1)
F1	0.47032 (19)	0.52149 (9)	0.89774 (16)	0.0561 (5)	0.557 (2)
F1X	0.56321 (18)	0.57786 (10)	0.4987 (2)	0.0389 (5)	0.443 (2)
F2	0.63567 (10)	0.50352 (5)	0.75745 (11)	0.0597 (3)	
O1	0.13830 (10)	0.74131 (4)	0.30771 (8)	0.03033 (18)	
N1	0.16602 (10)	0.70449 (5)	0.54187 (9)	0.02392 (17)	
C1	−0.28769 (13)	0.78053 (6)	0.36744 (13)	0.0319 (2)	
H1A	−0.2742	0.8252	0.4238	0.038*	
C2	−0.42202 (15)	0.74209 (7)	0.31632 (16)	0.0392 (3)	
H2A	−0.4989	0.7598	0.3397	0.047*	
C3	−0.44453 (15)	0.67783 (8)	0.23120 (16)	0.0412 (3)	
H3A	−0.5370	0.6516	0.1954	0.049*	
C4	−0.33179 (16)	0.65211 (7)	0.19871 (15)	0.0409 (3)	
H4A	−0.3472	0.6082	0.1397	0.049*	
C5	−0.19550 (14)	0.69013 (7)	0.25202 (13)	0.0334 (2)	
H5A	−0.1183	0.6718	0.2298	0.040*	
C6	−0.17167 (12)	0.75480 (6)	0.33758 (11)	0.02586 (19)	
C7	−0.02249 (12)	0.79671 (6)	0.40525 (10)	0.02398 (18)	
H7A	0.0016	0.8095	0.5132	0.029*	
C8	−0.02398 (12)	0.86899 (5)	0.32524 (11)	0.02398 (18)	
C9	0.06364 (13)	0.92810 (6)	0.41162 (12)	0.0299 (2)	
H9A	0.1219	0.9227	0.5192	0.036*	
C10	0.06666 (15)	0.99493 (6)	0.34203 (14)	0.0344 (2)	
H10A	0.1272	1.0348	0.4020	0.041*	
C11	−0.01879 (15)	1.00341 (6)	0.18504 (14)	0.0335 (2)	
H11A	−0.0178	1.0492	0.1374	0.040*	
C12	−0.10549 (13)	0.94485 (6)	0.09827 (13)	0.0315 (2)	
H12A	−0.1634	0.9505	−0.0093	0.038*	
C13	−0.10860 (12)	0.87771 (6)	0.16718 (11)	0.02652 (19)	
H13A	−0.1683	0.8378	0.1066	0.032*	
C14	0.10303 (12)	0.74572 (5)	0.41253 (10)	0.02300 (18)	
C15	0.28155 (11)	0.65108 (5)	0.58492 (10)	0.02453 (19)	
C16	0.31134 (13)	0.61006 (6)	0.71693 (11)	0.0316 (2)	
H16A	0.2508	0.6162	0.7679	0.038*	
C17	0.43000 (15)	0.56061 (7)	0.77189 (13)	0.0407 (3)	
H17A	0.4512	0.5327	0.8616	0.049*	0.443 (2)
C18	0.51753 (15)	0.55115 (7)	0.69909 (15)	0.0426 (3)	
C19	0.48590 (14)	0.58994 (7)	0.56743 (14)	0.0370 (3)	
H19A	0.5457	0.5822	0.5160	0.044*	0.557 (2)
C20	0.36784 (12)	0.64020 (6)	0.50857 (12)	0.0284 (2)	
H20A	0.3463	0.6668	0.4173	0.034*	
H1N1	0.1318 (17)	0.7138 (8)	0.6086 (17)	0.038 (4)*	

### Atomic displacement parameters (Å^2^)

**Table:**

	*U*^11^	*U*^22^	*U*^33^	*U*^12^	*U*^13^	*U*^23^
F1	0.0638 (10)	0.0395 (8)	0.0371 (7)	−0.0028 (7)	−0.0014 (7)	0.0203 (6)
F1X	0.0291 (8)	0.0390 (9)	0.0512 (10)	0.0090 (7)	0.0206 (7)	0.0087 (7)
F2	0.0463 (5)	0.0449 (5)	0.0598 (5)	0.0190 (4)	−0.0003 (4)	0.0138 (4)
O1	0.0440 (4)	0.0325 (4)	0.0208 (3)	0.0134 (3)	0.0203 (3)	0.0071 (3)
N1	0.0304 (4)	0.0267 (4)	0.0160 (3)	0.0008 (3)	0.0118 (3)	0.0027 (3)
C1	0.0358 (6)	0.0282 (5)	0.0345 (5)	0.0092 (4)	0.0184 (4)	0.0082 (4)
C2	0.0332 (6)	0.0376 (6)	0.0483 (7)	0.0096 (5)	0.0198 (5)	0.0154 (5)
C3	0.0347 (6)	0.0402 (7)	0.0429 (6)	−0.0029 (5)	0.0126 (5)	0.0131 (5)
C4	0.0485 (7)	0.0336 (6)	0.0398 (6)	−0.0085 (5)	0.0195 (6)	−0.0023 (5)
C5	0.0405 (6)	0.0308 (5)	0.0332 (5)	−0.0027 (5)	0.0205 (5)	−0.0032 (4)
C6	0.0326 (5)	0.0244 (4)	0.0223 (4)	0.0032 (4)	0.0140 (4)	0.0047 (3)
C7	0.0324 (5)	0.0243 (4)	0.0185 (4)	0.0036 (4)	0.0144 (3)	−0.0003 (3)
C8	0.0311 (5)	0.0225 (4)	0.0228 (4)	0.0043 (4)	0.0161 (4)	−0.0008 (3)
C9	0.0409 (6)	0.0266 (5)	0.0263 (4)	−0.0002 (4)	0.0189 (4)	−0.0054 (4)
C10	0.0471 (7)	0.0241 (5)	0.0403 (6)	−0.0028 (5)	0.0271 (5)	−0.0073 (4)
C11	0.0433 (6)	0.0236 (5)	0.0432 (6)	0.0061 (4)	0.0280 (5)	0.0052 (4)
C12	0.0350 (5)	0.0312 (5)	0.0304 (5)	0.0059 (4)	0.0167 (4)	0.0077 (4)
C13	0.0312 (5)	0.0257 (5)	0.0241 (4)	0.0030 (4)	0.0139 (4)	0.0016 (3)
C14	0.0307 (5)	0.0227 (4)	0.0169 (3)	0.0018 (4)	0.0120 (3)	0.0009 (3)
C15	0.0281 (5)	0.0220 (4)	0.0182 (3)	−0.0023 (4)	0.0059 (3)	0.0023 (3)
C16	0.0382 (6)	0.0274 (5)	0.0215 (4)	−0.0067 (4)	0.0069 (4)	0.0051 (4)
C17	0.0451 (7)	0.0289 (6)	0.0278 (5)	−0.0035 (5)	−0.0010 (5)	0.0100 (4)
C18	0.0370 (6)	0.0289 (6)	0.0396 (6)	0.0062 (5)	−0.0020 (5)	0.0058 (5)
C19	0.0332 (6)	0.0319 (6)	0.0374 (6)	0.0046 (5)	0.0086 (5)	0.0013 (4)
C20	0.0304 (5)	0.0270 (5)	0.0244 (4)	0.0035 (4)	0.0094 (4)	0.0035 (3)

### Geometric parameters (Å, º)

**Table:**

F1—C17	1.3157 (17)	C8—C9	1.3938 (15)
F1X—C19	1.253 (2)	C8—C13	1.3967 (13)
F2—C18	1.3561 (14)	C9—C10	1.3913 (16)
O1—C14	1.2316 (11)	C9—H9A	0.9500
N1—C14	1.3533 (12)	C10—C11	1.3883 (18)
N1—C15	1.4107 (14)	C10—H10A	0.9500
N1—H1N1	0.881 (15)	C11—C12	1.3843 (17)
C1—C2	1.3839 (18)	C11—H11A	0.9500
C1—C6	1.3960 (15)	C12—C13	1.3931 (15)
C1—H1A	0.9500	C12—H12A	0.9500
C2—C3	1.385 (2)	C13—H13A	0.9500
C2—H2A	0.9500	C15—C20	1.3882 (15)
C3—C4	1.381 (2)	C15—C16	1.4006 (13)
C3—H3A	0.9500	C16—C17	1.3802 (18)
C4—C5	1.3944 (18)	C16—H16A	0.9500
C4—H4A	0.9500	C17—C18	1.367 (2)
C5—C6	1.3918 (15)	C17—H17A	0.9500
C5—H5A	0.9500	C18—C19	1.3733 (18)
C6—C7	1.5253 (15)	C19—C20	1.3863 (16)
C7—C8	1.5172 (14)	C19—H19A	0.9500
C7—C14	1.5283 (14)	C20—H20A	0.9500
C7—H7A	1.0000		
			
C14—N1—C15	128.51 (8)	C9—C10—H10A	120.0
C14—N1—H1N1	114.9 (10)	C12—C11—C10	119.66 (10)
C15—N1—H1N1	116.6 (10)	C12—C11—H11A	120.2
C2—C1—C6	121.09 (11)	C10—C11—H11A	120.2
C2—C1—H1A	119.5	C11—C12—C13	120.60 (10)
C6—C1—H1A	119.5	C11—C12—H12A	119.7
C1—C2—C3	120.10 (12)	C13—C12—H12A	119.7
C1—C2—H2A	120.0	C12—C13—C8	120.06 (10)
C3—C2—H2A	120.0	C12—C13—H13A	120.0
C4—C3—C2	119.61 (12)	C8—C13—H13A	120.0
C4—C3—H3A	120.2	O1—C14—N1	124.08 (9)
C2—C3—H3A	120.2	O1—C14—C7	122.62 (8)
C3—C4—C5	120.39 (12)	N1—C14—C7	113.25 (8)
C3—C4—H4A	119.8	C20—C15—C16	120.11 (10)
C5—C4—H4A	119.8	C20—C15—N1	123.88 (8)
C6—C5—C4	120.49 (11)	C16—C15—N1	115.95 (9)
C6—C5—H5A	119.8	C17—C16—C15	119.03 (11)
C4—C5—H5A	119.8	C17—C16—H16A	120.5
C5—C6—C1	118.30 (11)	C15—C16—H16A	120.5
C5—C6—C7	122.82 (10)	F1—C17—C18	115.35 (14)
C1—C6—C7	118.83 (9)	F1—C17—C16	123.65 (15)
C8—C7—C6	114.95 (8)	C18—C17—C16	120.96 (11)
C8—C7—C14	110.80 (8)	C18—C17—H17A	119.5
C6—C7—C14	109.82 (8)	C16—C17—H17A	119.5
C8—C7—H7A	107.0	F2—C18—C17	119.82 (12)
C6—C7—H7A	107.0	F2—C18—C19	120.18 (14)
C14—C7—H7A	107.0	C17—C18—C19	120.00 (11)
C9—C8—C13	118.95 (9)	F1X—C19—C18	118.92 (13)
C9—C8—C7	119.05 (9)	F1X—C19—C20	120.23 (13)
C13—C8—C7	121.99 (9)	C18—C19—C20	120.82 (12)
C10—C9—C8	120.71 (10)	C18—C19—H19A	119.6
C10—C9—H9A	119.6	C20—C19—H19A	119.6
C8—C9—H9A	119.6	C19—C20—C15	119.03 (10)
C11—C10—C9	120.01 (11)	C19—C20—H20A	120.5
C11—C10—H10A	120.0	C15—C20—H20A	120.5
			
C6—C1—C2—C3	−1.41 (17)	C15—N1—C14—O1	1.15 (17)
C1—C2—C3—C4	0.48 (18)	C15—N1—C14—C7	178.67 (9)
C2—C3—C4—C5	0.48 (19)	C8—C7—C14—O1	−37.01 (13)
C3—C4—C5—C6	−0.52 (19)	C6—C7—C14—O1	91.06 (11)
C4—C5—C6—C1	−0.38 (16)	C8—C7—C14—N1	145.43 (9)
C4—C5—C6—C7	177.03 (10)	C6—C7—C14—N1	−86.51 (10)
C2—C1—C6—C5	1.35 (16)	C14—N1—C15—C20	10.68 (16)
C2—C1—C6—C7	−176.17 (10)	C14—N1—C15—C16	−172.12 (10)
C5—C6—C7—C8	106.72 (11)	C20—C15—C16—C17	1.90 (16)
C1—C6—C7—C8	−75.88 (11)	N1—C15—C16—C17	−175.41 (10)
C5—C6—C7—C14	−19.02 (12)	C15—C16—C17—F1	177.53 (13)
C1—C6—C7—C14	158.38 (9)	C15—C16—C17—C18	−0.26 (18)
C6—C7—C8—C9	145.85 (9)	F1—C17—C18—F2	0.46 (19)
C14—C7—C8—C9	−88.93 (10)	C16—C17—C18—F2	178.43 (11)
C6—C7—C8—C13	−35.24 (13)	F1—C17—C18—C19	−179.40 (13)
C14—C7—C8—C13	89.99 (11)	C16—C17—C18—C19	−1.44 (19)
C13—C8—C9—C10	0.23 (16)	F2—C18—C19—F1X	3.4 (2)
C7—C8—C9—C10	179.18 (10)	C17—C18—C19—F1X	−176.70 (14)
C8—C9—C10—C11	0.33 (17)	F2—C18—C19—C20	−178.36 (11)
C9—C10—C11—C12	−0.68 (17)	C17—C18—C19—C20	1.50 (19)
C10—C11—C12—C13	0.46 (17)	F1X—C19—C20—C15	178.32 (14)
C11—C12—C13—C8	0.11 (16)	C18—C19—C20—C15	0.14 (18)
C9—C8—C13—C12	−0.45 (15)	C16—C15—C20—C19	−1.84 (16)
C7—C8—C13—C12	−179.37 (9)	N1—C15—C20—C19	175.24 (10)

### Hydrogen-bond geometry (Å, º)

Cg1 and Cg2 are the centroids of the C1–C6 and C8–C13 rings, respectively.

**Table:**

*D*—H···*A*	*D*—H	H···*A*	*D*···*A*	*D*—H···*A*
N1—H1*N*1···O1^i^	0.881 (15)	2.088 (16)	2.9134 (11)	155.7 (14)
C13—H13*A*···*Cg*1^ii^	0.95	2.98	3.7633 (11)	140
C16—H16*A*···*Cg*2^iii^	0.95	2.82	3.5998 (15)	140
C20—H20*A*···O1	0.95	2.29	2.8878 (14)	120

Symmetry codes: (i) *x*, −*y*+3/2, *z*+1/2; (ii) *x*, −*y*+1/2, *z*−3/2; (iii) *x*, −*y*+1/2, *z*−1/2.
